# Lung involvement in childhood onset granulomatosis with polyangiitis

**DOI:** 10.1186/s12969-017-0150-8

**Published:** 2017-04-14

**Authors:** Giovanni Filocamo, Sofia Torreggiani, Carlo Agostoni, Susanna Esposito

**Affiliations:** 1 Pediatric Rheumatology, Fondazione IRCCS Ca’ Granda Ospedale Maggiore Policlinico, via Commenda 9, 20122 Milan, Italy; 2 Fondazione IRCCS Ca’ Granda Ospedale Maggiore Policlinico, Università degli Studi di Milano, Milan, Italy; 3 Pediatric Clinic, Department of Surgical and Biomedical Sciences, Università degli Studi di Perugia, Perugia, Italy

**Keywords:** Granulomatosis with polyangiitis, Wegener granulomatosis, Childhood, Lung, Pulmonary

## Abstract

Granulomatosis with polyangiitis is an ANCA-associated systemic vasculitis with a low incidence in the pediatric population. Lung involvement is a common manifestation in children affected by granulomatosis with polyangiitis, both at disease’s onset and during flares. Its severity is variable, ranging from asymptomatic pulmonary lesions to dramatic life-threatening clinical presentations such as diffuse alveolar haemorrhage. Several radiologic findings have been described, but the most frequent abnormalities detected are nodular lesions and fixed infiltrates. Interstitial involvement, pleural disease and pulmonary embolism are less common. Histology may show necrotizing or granulomatous vasculitis of small arteries and veins of the lung, but since typical features may be patchy, the site for lung biopsy should be carefully chosen with the help of imaging techniques such as computed tomography. Bronchoalveolar lavage is helpful to confirm the diagnosis of alveolar haemorrhage. Pulmonary function tests are frequently altered, showing a reduction in the diffusion capacity for carbon monoxide, which can be associated with obstructive abnormalities related to airway stenosis. Nodular lung lesions tend to regress with immunosuppressive therapy, but lung disease may also require second line treatments such as plasmapheresis. In cases of massive diffuse alveolar haemorrhage, ventilator support is crucial in the management of the patient.

## Background

Granulomatosis with polyangiitis (GPA), formerly known as Wegener’s granulomatosis [1], is an idiopathic vasculitis of medium and small arteries, characterized by necrotizing granulomatous inflammation of the respiratory tract with necrotizing, pauci-immune glomerulonephritis; vasculitis frequently involves also other organs. GPA is a member of a family of vasculitides associated with positive anti-neutrophil cytoplasmic antibodies (ANCA). In GPA, the pattern of autoantibody staining of ethanol-fixed neutrophils is typically cytoplasmic (c-ANCA), rather than perinuclear (p-ANCA), due to the presence of antibodies against proteinase 3, which is a constituent of the azurophilic granules of the neutrophil [2, 3].

The family of ANCA-associated vasculitides (AAV) also includes eosinophilic granulomatosis with polyangiitis (EGPA, previously known as Churg-Strauss syndrome) and microscopic polyangiitis (MPA). The clinical spectrum of vasculitides is broad; the lack of pathognomonic features and the presence of overlapping clinical manifestations makes diagnosis challenging, especially in discriminating one form from one another. In 1990 the American College of Rheumatology (ACR) published the classification criteria for seven vasculitides, including GPA and EGPA [4–7]. However, because of the poor performance of ACR criteria in classifying children with vasculitis, the EULAR/PRINTO/PRES criteria were developed using pediatric data. The EULAR/PRINTO/PRES criteria for GPA (Table 1) showed improved sensitivity compared to the adult-based ACR criteria (93% vs 83%) [8–10]. However, both classification criteria did not include MPA, which had its specific definition thanks to the Chapel Hill Consensuses Conference (CHCC) [11]. It is therefore possible that cases classified as GPA according to ACR criteria may be described as MPA using the CHCC definition. In 2007 the European Medicines Agency (EMA) endorsed a classification algorithm to classify patients with a mutually exclusive diagnosis (EGPA, GPA, MPA, or polyarteritis nodosa) [12]. The EMA classification algorithm showed to be a promising tool to uniquely diagnose children with either GPA or MPA [13].

**Table 1:** EULAR/PRINTO/PRES criteria for childhood granulomatosis with polyangiitis [6]

1. Histopathology	Granulomatous inflammation within the wall of an artery or in the perivascular or extravascular area
2. Upper airway involvement	Chronic purulent or bloody nasal discharge or recurrent epistaxis/crusts/granulomata
	Nasal septum perforation or saddle nose deformity
	Chronic or recurrent sinus inflammation
3. Laryngo-tracheo-bronchial involvement	Subglottic, tracheal or bronchial stenosis
4. Pulmonary involvement	Chest x-ray or CT showing the presence of nodules, cavities or fixed infiltrates
5. ANCA	ANCA positivity by immunofluorescence or by ELISA (MPO/p or PR3/c ANCA)
6. Renal involvement	Proteinuria >0.3 g/24 h or >30 mmol/mg of urine albumin/creatinine ratio on a spot morning sample
	Haematuria or red blood cell casts: >5 red blood cells/high power field or red blood cells casts in the urinary sediment or ≥2+ on dipstick
	Necrotising pauci-immune glomerulonephritis

The presence of at least three of the six criteria has a sensitivity of 93.3% and a specificity of 99.2% in confirming the diagnosis

GPA onset usually occurs between 45 and 60 years of age, with a peak in the sixth decade [2, 14, 15], but in a small proportion of cases (3.3–7%) it may affect also children and adolescents. Incidence of juvenile onset GPA is not well known but estimates range from 0.02 to 0.64 per 100,000 persons per year [16, 17]. Pediatric GPA is usually diagnosed in adolescence [18–33], with a median age at onset of 11.6 years and a median age at diagnosis of 14 years, and presents a female predominance with a male to female ratio of 1:2.1 [18].

The most common clinical manifestations of childhood GPA at onset are related to upper airway involvement (82%), nephropathy (65%) and lower respiratory tract disease (61%). This characteristic triad is frequently associated with systemic symptoms (73%) [18]. Although the main aim of this review is to present lung involvement in GPA, upper airway disease is briefly summarized below in the next paragraph, as it affects respiratory findings in patients with GPA.

According to the EULAR/PRINTO/PRES criteria, upper airway involvement can be assessed in presence of chronic or bloody nasal discharge, recurrent epistaxis/crusts/granulomata, septal perforation or sinus inflammation [9]. Sinonasal disease is a common presenting feature of GPA in children, which should be distinguished from infectious or allergic rhinitis or rhinosinusitis [18, 24, 32]. Longstanding disease can damage nasal cartilage, leading to septal perforation and saddle-nose deformity. Other manifestations of ear, nose and throat involvement include otitis, mastoiditis, oral ulcers or granulomata, mucocele, hearing loss and subglottic stenosis [19, 21, 22, 24, 33]. Young patients under 20 years of age are more prone to develop tracheal involvement [34]; subglottic stenosis was reported to complicate childhood GPA five times as often as in adults [17]. Therefore, laryngo-tracheo-bronchial stenosis was included in the pediatric EULAR/PRINTO/PRES criteria [9]. In a cohort of 28 pediatric patient affected by GPA, airway stenosis was detected in 36% of patients at diagnosis and in 50% during follow-up, with involvement of the tracheobronchial tree in a third of cases [35]. The common symptoms caused by tracheobronchial involvement include hoarseness, cough, dyspnea, stridor, and wheezing [36–38].

## Lung involvement in childhood-onset GPA

### Prevalence and clinical features

Lung involvement is a frequent feature of GPA. Main features of lung involvement in children with GPA are displayed in Table 2. X-ray evidence of fixed pulmonary infiltrates, nodules, or cavitations for more than 1 month essentially precludes a diagnosis of MPA according to the EMA algorithm to classify vasculitis [12]. MPA is a necrotizing vasculitis with few or no immune deposits, predominantly affecting small vessels (capillaries, venules, or arterioles) and more rarely small- and medium-sized arteries [11]. The limited available literature suggests that genetic, pathophysiological and prognostic differences exist between MPA and GPA, but distinguishing between these diseases is challenging because of overlapping clinical features. The absence of granulomata and sparing of the upper respiratory tract are features of MPA, in which necrotizing glomerulonephritis and pulmonary capillaritis are common. These features help to distinguish MPA from GPA [11].

**Table 2:** Frequency and main features of lung involvement in patients affected with GPA in pediatric series

First author and year of publication	Number of patients	Median age at diagnosis (range)	Female	Lung involvement (%)	Hemopstysis/Alveolar haemorrage	Dyspnea	Chronic cough	Pleural effusion/thickening	Lung nodules or cavity	Lung infiltrate
Tahghighi 2013 [23]	11	11 (6–15)	5	7 (63,6%)	2	2	N.R.	1	N.R.	N.R.
Iudici 2015 [25]	25	14 (2–17)	18	17 (68%)	3	3	N.R.	N.R.	7	6
Sacri 2015 [26]	28	12,8 (10,1–14,6)	21	19 (67,8%)	12	N.R.	N.R.	1	7	16
Bohm 2014 [30]	56	N.R.	38	44 (78,5%)	14/55	N.R.	N.R.	7	17	26/55
Kosalka 2014 [31]	9	N.R.	6	8 (88,9%)	4	N.R.	N.R.	2	4	2
Cabral 2016 [32]	183	14 (2–18)	113	136 (74,3%)	76	15	99	25	97	64
Belostotsky 2002 [33]	17	N.R.	13	14 (82,3%)	3	4	9	N.R.	2	2
Akikusa 2007 [22]	25	14,5 (8,7–17,1)	20	21 (84%)	12	N.R.	N.R.	2	13	6
Arulkumaran 2011 [27]	7	N.R.	5	5 (71,4%)	2	N.R.	N.R.	N.R.	N.R.	N.R.
Wong 1998 [28]	12	N.R.	8	7 (58,3%)	3	N.R.	N.R.	N.R.	N.R.	N.R.

*N.R.* not reported

Lung disease is frequently the presenting clinical manifestation of GPA, however it can sometimes develop later during the disease course. Pulmonary involvement is frequent during disease’s flares, also in patients who did not present lung disease at GPA onset [22].

In the ARChiVe (A Registry for Children with Vasculitis) cohort including 65 children affected by GPA, shortness of breath was reported in 58.5% of cases and chronic cough in 52.3% [20]. In a subsequent study on the ARChiVe cohort, including 183 patients with childhood-onset GPA, pulmonary involvement was detected in 74% of patients. Overall, 54% of patients presented chronic cough, while only 8% showed wheezing or expiratory dyspnea. Massive haemoptysis or alveolar haemorrhage was reported in 42% of patients. Chest imaging was abnormal in 89% of patients, showing nodules in 54% of cases, fixed pulmonary infiltrates in 36% and cavitations in 21%; other findings such as fibrosis, septal thickening and pneumothorax were detected in less than 10% of cases. Pleurisy affected 14% of patients: 12% of patients had respiratory failure and 22% required supplemental oxygen. In 31 patients lung biopsy was performed: in 77% of cases biopsy findings confirmed the presence of vasculitis or were consistent with features of vasculitis [32].

In the meta-analysis published by Iudici et al., lower respiratory tract involvement has a prevalence of 61% (95% confidence interval [CI] 48–74%), with hemoptysis/alveolar haemorrage occurring in 16% of patients and lung nodules identified in 10% of patients [18].

It should be noted that Rottem et al. reported that 41% of radiographic abnormalities found in patients with childhood GPA were observed in absence of symptoms. [21]

Manifestations of lung involvement during flares are variable, but in a pediatric series of 25 patients, alveolar haemorrhage was reported to be the least common condition observed [22]. Considering mortality in childhood GPA, together with infections, respiratory complications (including pulmonary haemorrhage and chronic lung disease) were the most common causes of death [25, 32].

#### Nodules

Nodular lesions are the most common pulmonary radiologic finding in GPA, in both adults and children [22, 32, 39]. Their presence is not necessarily associated with clinical signs and symptoms. [21] They consist of granulomatous inflammation and necrosis. In children both multiple and single nodular lesions have been described; on chest radiography, their size may vary from 1 to 4 cm [29, 39–41]. In adult patients, nodules are usually multiple but in a number not greater than ten, with a size ranging from 1 to 10 cm [39]. Computed tomography (CT) shows also smaller nodules and can detect intranodular cavitation, that cannot be seen on chest x rays. Without treatment, nodules increase in number and size and tend to cavitate.

Pulmonary nodules usually regress with therapy in weeks or months, but local scarring is possible. Persistent nodules may indicate active disease, but could also be due to cicatricial residues in patients with inactive disease [39, 42]. The absence of regression or the extension of the lesion in a patient who is receiving immunosuppressive treatment may also be caused by a bacterial superinfection [43].

#### Diffuse alveolar haemorrhage

Diffuse alveolar haemorrhage is a life-threatening condition which presents with haemoptysis, anemia, and diffuse alveolar infiltrates leading to respiratory failure. The clinical triad is often incomplete, in particular hemoptysis may be absent in about a third of cases [44]. Hypocomplementemia at the time of diagnosis was recognized as a risk factor for alveolar haemorrage [45].

Chest radiography shows a bilateral alveolar filling pattern, involving perihilar and basal lung fields. Bronchial fibroscopy shows a peripheral diffuse bleeding, and is required to exclude a localized cause of hemorrage. Bronchoalveolar lavage (BAL) allows detection of blood red cells if the haemorrhage is acute or siderophages if it is chronic; furthermore, it is useful in ruling out infections, that may be suspected also due to the presence of constitutional symptoms such as fever [39].

The underlying histologic findings of diffuse alveolar haemorrage are neutrophilic infiltration and fibrinoid necrosis of the alveolar and capillary walls [46].

A study in the adult population showed that in patients with alveolar haemorrage due to AAV, the degree of hypoxemia upon presentation was the most important predictor of respiratory failure; a higher disease activity score, a higher percentage of neutrophils in the BAL fluid and higher C reactive protein levels were also associated with respiratory failure [47].

#### Other manifestations

Pulmonary fibrosis is uncommon in patients with antineutrophil cytoplasmic antibodies-associated vasculitis and mainly occurs in microscopic polyangiitis in association with anti-MPO antibodies; nonetheless, it was described in adult patients affected with GPA [48–51] and also in pediatric patients [32]. Interstitial involvement in a treated patient should induce the suspicion of a iatrogenic pneumopathy [39, 52].

Pleural involvement is present in a minority of patients, and may consist of pleural thickening, pneumothorax or pyopneumothorax, which can be caused by the rupture of a peripheral cavitated lesion into the pleural cavity.

It should be remembered that GPA is associated with an increased risk of venous thromboembolic events in adults and children: although in a small proportion of cases, also pulmonary embolic disease has been described in patients with childhood onset GPA, both at disease presentation and during follow-up [22, 53, 54].

Necrotizing granulomas not only alter the normal alveolar architecture progressively impairing the respiratory function, but may also cause bronchial stenosis. Multilevel airway stenosis was described in about one third of adult patients, in particular 17% had mid/distal tracheal stenosis and 11% had bronchial stenosis [55]. In children affected by GPA, tracheobronchial stenosis was detected in 17% of cases [35].

### Histological findings

The simplest approach to collect tissue for histological analysis is transbronchial biopsy, but blind transbronchial biopsy of lung tissue is rarely informative because of the focal distribution of the inflammatory lesions in GPA. Open-lung biopsy has a higher accuracy, but also more significant morbidity [56–58]. Computed tomography may help in choosing the best site for tissue sampling, in order to increase the yield of transbronchial biopsy; lesions with little necrotic component should be preferred. In adults, image-guided automated core needle biopsy was even used to obtain histological proof of disease activity and guide therapy [59].

Necrotizing or granulomatous vasculitis may be found in small arteries and veins of the lung. The lumen of the vessels may be occluded by inflammatory infiltrate or by a thrombus [60]. Granulomas show central necrosis surrounded by histiocytes, lymphocytes and giant cells; also eosinophils may be present but in small proportion. In some cases nonspecific inflammation may be found [17].

Histology in pulmonary haemorrhage generally shows pulmonary capillaritis, which consists of interstitial neutrophilic predominant infiltration, fibrinoid necrosis of the alveolar and capillary walls and leukocytoclasis, with subsequent loss of the integrity of the alveolar capillary membrane [46].

### Radiologic findings

The typical lower respiratory tract involvement of GPA is demonstrated by radiologic findings of lung nodules, cavities or fixed infiltrates. In adults the most common radiological presentation is represented by multiple nodules randomly distributed throughout the lung, of variable size as mentioned above. They range from well to poorly defined and may be distributed along bronchovascular bundles. In approximately a half of cases, some of the nodules demonstrate cavitation, which is best seen by CT [61, 62].

**Fig. 1:**
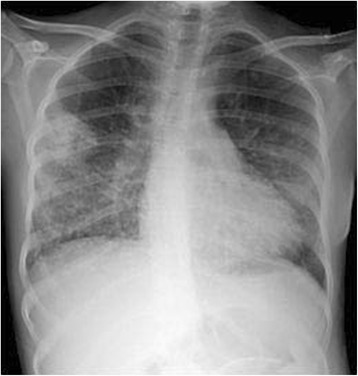
Chest x ray in a 16-year old girl affected by granulomatosis with polyangiitis (GPA). The image shows multiple nodular lesions together with diffuse opacities in the lower and middle regions of the lung

Presence of nodules was the most frequent observation also in pediatric patients (Fig. 1) [22, 32]. However, in a small cohort of children, diffuse interstitial and alveolar opacities attributable to pulmonary hemorrhage were more common than nodular lesions [41]. Both nodules and regions of consolidation can be surrounded by haemorrhage, which in some cases dominates the radiographic appearance. Unfortunately, the radiographic appearance of GPA is very variable and not specific, making a diagnosis by imaging alone often difficult [39].

**Fig. 2:**
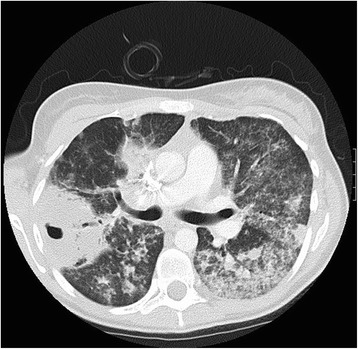
High-resolution chest computed tomography in a 16-year old girl affected by granulomatosis with polyangiitis (GPA). The image shows multiple nodules and regions of consolidation of variable size, irregularly marginated with peribronchovascular distribution. Cavitations are demonstrated in several nodules, the largest in the right lung (6.5×4.5 cm). Diffuse alveolar opacities are consequence of haemorrhages

Conventional chest X rays can easily detect nodules and masses, fixed infiltrates, cavitations, pleural effusions and pneumothoraces, while small nodules, linear opacities, focal infiltrates and fluffy perivascular densities are best identified using high-resolution computed tomography (HRCT) [17] (Fig. 2). Bilateral perihilar and basal infiltrates of alveolar haemorrhage may resemble pulmonary oedema, but differential diagnosis can be based on the absence of cardiomegaly and signs of venous hypertension. Following acute diffuse alveolar haemorrage, a radiographic pattern of septal thickening, known as crazy-paving, can appear [62].

The most common HRCT abnormalities are lung nodules, usually multiple and bilateral which tend to increase with disease progression. They can range from few millimeters to >10 cm in diameter and become cavitated. Cavities are usually thick walled and characterized by an irregular inner margin and absent calcification. The nodular lesions are often related to the vessels, and they tend to involve mainly the subpleural regions but have no predilection for the upper or lower lung zones. Haemorrhage may result in a HRCT finding consisting of an edge of ground glass opacity surrounding the lung lesion, known as halo sign [62, 63]. HRCT could be used also to identify active disease: ground-glass opacities, cavitations and masses measuring more than 3 cm tend to correspond to active disease; while non-cavitated small nodules and linear opacities can also represent cicatricial lesions [64].

18-F-fluorodeoxyglucose positron emission tomography/computed tomography scans have been used to evaluate global disease distribution and to identify active lesions suitable for biopsy [65, 66].

### Pulmonary function tests

When tested for pulmonary function, patients affected by GPA most commonly show airflow obstruction, often in association with a reduced diffusion capacity of the lung for carbon monoxide. Reduction of lung volumes has also been described [67, 68]. In a pediatric series, of the 35 patients who performed pulmonary function tests, 27 had abnormal results, with obstructive and restrictive abnormalities detected in the same proportion of cases [20]. Sixty-seven patients of the ARChiVe cohort performed pulmonary function tests, which were abnormal in 61% of cases [32]. Since pulmonary involvement in GPA may be initially asymptomatic, it should be investigated not only radiographically, but also with pulmonary function testing. The first sign of pulmonary haemorrhage may be an alteration in the diffusion capacity of the lung for carbon monoxide [21, 57, 69]. In patients with subglottic stenosis, the flow-volume curve shows a flattening in both inspiratory and expiratory phase, consistent with an extrathoracic airway obstruction [70].

### Differential diagnosis

In the presence of lung disease, concomitant upper airway and kidney involvement can guide the clinician towards GPA diagnosis. Other conditions associated with pulmonary-renal syndrome, such as Goodpasture’s syndrome, polyarteritis nodosa, systemic lupus eythematosus or mixed connective tissue disease, should be excluded. Laboratory evidence of positive ANCA, in particular c-ANCA or PR3-ANCA, and a compatible kidney biopsy, are supportive findings of GPA diagnosis. Lung involvement of ulcerative colitis associated with ANCA positivity may also mimic GPA [71].

Considering nodular lung lesions, neoplastic disease and sarcoidosis should also be suspected. In children, also chronic granulomatous disease has to be considered [72]. Other causes of alveolar haemorrhage described in the pediatric population are idiopathic pulmonary haemosiderosis and childhood onset systemic lupus erythematosus [73–75].

Another important differential diagnosis is with infectious processes, in particular mycobacterial, micotic or helmintic infections [76]. Diffuse alveolar haemorrhage has been described as a consequence of pulmonary histoplasmosis [77]. It should also be remembered that lung disease in GPA patients may also be due to concomitant infections, in particular *Pneumocystis jirovecii* infection in the immunocompromised patient [78, 79].

## Treatment

Treatment requires a remission induction phase, followed by maintenance therapy. Standard treatment of GPA has primarily consisted of glucocorticoids and cyclophosphamide. Several other immunosuppressive agents have been used, in monotherapy or in combination with glucocorticoids, including methotrexate, azathioprine, mycophenolate, cyclosporine, colchicines, etanercept, infliximab, adalimumab, rituximab. Remission induction is mainly based on the use of glucocorticoids and cyclophosphamide; cyclophosphamide can be administered either orally or with intravenous pulses and should be withdrawn when remission is achieved. Remission maintenance therapy, which should last at least 18–24 months, is usually based on the introduction of azathioprine or methotrexate, with concomitant tapering of glucocorticoids [18, 19, 21, 80–84].

Other therapeutic strategies have been used in severe and refractory cases. Intravenous immunoglobulin administration has been used as adjunctive therapy in AAV patients with refractory or relapsing disease, even if 11,9% of patients presented serious adverse events [85]. In severe cases of alveolar haemorrage, together with aggressive immunosuppressive therapy, plasmapheresis has been used [86]. The use of plasma exchange in patients with creatinine levels > 5,8 mg/dl was proved to be more effective than intravenous methylprednisolone in preventing end-stage renal disease at 12 months, but no difference was observed in survival rates and incidence of adverse events [87]. Even if plasma exchange is usually reserved for refractory disease and life-threatening conditions, it has been proposed also for induction of remission in less severe cases [88]. The PEXIVAS study will better define the benefits of plasma exchange in AAV. [89] Kidney involvement may require dialysis and kidney transplant [90].

So far, no clinical trial has been conducted in the pediatric population and no pediatric specific recommendations are available. Pediatric guidelines for GPA will be hopefully developed by the SHARE project, whose aim is to provide recommendations for the care of children and young adults with rheumatic diseases, including vasculitis [91]. At the moment, therapeutic management is guided by data extrapolated from adult studies. Guidelines for the treatment of AAV in adult patients have recently been published [92, 93].

Of note, both British and EULAR recommendations include rituximab, an anti-CD20 monoclonal antibody, which has proved to be a promising new therapeutic option for AAV both in remission induction and in remission maintenance [92–96]. The RAVE trial, which included AAV patients older than 15 years, showed that rituximab was not inferior to cyclophosphamide in achieving remission induction and was more effective for relapsing disease; better response to rituximab was observed in anti-PR3 positive patients [94]. Rituximab should be considered as a therapeutic option for remission induction also in children, since steroid sparing in pediatric age is mandatory and cyclophospahmide avoidance is especially desirable in young people at risk of infertility. In a pediatric series, 10 patients with primary systemic vasculitis, including 4 children with GPA, were treated with rituximab with a decrease in disease activity and in corticosteroid dose. Of 10 patients receiving rituximab, 3 presented adverse events: one patient with unclassified vasculitis had mild headache with second rituximab infusion; one patient with GPA developed paronychia 2 months after receiving rituximab treatment; one patient with GPA treated with both infliximab and rituximab presented, respectively 9 and 7 months after drug discontinuation, a Pseudomonas urinary tract infection and concurrent pneumonia, while he was also being administered concurrent immunosuppressive therapy with cyclosporine A for renal transplant [97]. At the moment, as long-term safety data on the use of rituximab in pediatric vasculitis are still missing, rituximab should be prescribed carefully, in refractory cases. A phase IIa international open-label trial is currently recruiting patients to evaluate the safety and pharmacokinetics of rituximab in children with severe GPA or MPA (PePRS study, NCT01750697), but mechanical ventilation due to alveolar hemorrhage represents an exclusion criterium for participation in the study. It should be noted that also the RAVE trial excluded patients with alveolar haemorrage requiring mechanical respiratory assistance upon enrollment [94].

A retrospective study analyzing a cohort of patients with alveolar haemorrage secondary to AAV, including also patients needing mechanical ventilation, showed that complete remission by 6 months was achieved in a higher proportion of cases with rituximab than with cyclophosphamide, even though the use of rituximab was not associated with a higher long-term survival rate. The same study could not demonstrate plasma exchange efficacy in addition to the standard remission induction therapy [47].

Lung disease can also necessitate the use of supportive measures. Pulmonary haemorrhage may require ventilator support in an intensive care unit setting or even extracorporeal membrane oxygenation [20, 98]. Akikusa and colleagues reported a median duration of intubation of 8 days in the subgroup of children requiring ventilation [22].

Airway lesions rarely respond satisfactorily to systemic therapy with cyclophosphamide, but rituximab appeared to be a more efficient therapy for tracheobronchial lesions [35, 99, 100]. In the presence of subglottic or tracheal involvement, surgical intervention is often needed to maintain a patent airway [101–103].

## Conclusion

Lung involvement is a common manifestation in children affected by GPA. Its severity is variable, ranging from asymptomatic pulmonary lesions to dramatic life-threatening clinical presentations such as diffuse alveolar haemorrhage. Several radiologic findings have been described, but the most frequent abnormalities detected are nodular lesions and fixed infiltrates. Nodular lung lesions tend to regress with immunosuppressive therapy, but lung disease may also require second line treatments. Rituximab may represent a promising treatment option also in pediatric patients, even if its efficacy and safety in children should be better studied. In cases of massive diffuse alveolar haemorrhage, ventilator support is crucial in the management of the patient.

## Abbreviations

ACR: American College of Rheumatology
CI: Confidence interval
CT: Computed tomography
GPA: Granulomatosis with polyangiitis
MPA: Microscopic polyangiitis
